# Meningitic *Escherichia coli*-Induced Interleukin-17A Facilitates Blood–Brain Barrier Disruption *via* Inhibiting Proteinase 3/Protease-Activated Receptor 2 Axis

**DOI:** 10.3389/fncel.2022.814867

**Published:** 2022-02-11

**Authors:** Bojie Xu, Jiaqi Chen, Jiyang Fu, Ruicheng Yang, Bo Yang, Dong Huo, Chen Tan, Huanchun Chen, Xiangru Wang

**Affiliations:** ^1^State Key Laboratory of Agricultural Microbiology, College of Veterinary Medicine, Huazhong Agricultural University, Wuhan, China; ^2^Key Laboratory of Preventive Veterinary Medicine in Hubei Province, The Cooperative Innovation Center for Sustainable Pig Production, Wuhan, China; ^3^Key Laboratory of Development of Veterinary Diagnostic Products, Ministry of Agriculture of the People’s Republic of China, Wuhan, China; ^4^International Research Center for Animal Disease, Ministry of Science and Technology of the People’s Republic of China, Wuhan, China

**Keywords:** IL-17A, *Escherichia coli* meningitis, blood-brain barrier, permeability, PRTN3, PAR-2

## Abstract

Bacterial meningitis is a life-threatening infectious disease with high morbidity and mortality worldwide, among which meningitic *Escherichia coli* is a common Gram-negative pathogenic bacterium causing meningitis. It can penetrate the blood–brain barrier (BBB), invoke local inflammatory responses and consequently disrupt the integrity of the BBB. Interleukin-17A (IL-17A) is recognized as a pro-inflammatory cytokine that is released during meningitic *E. coli* infection. It has been reported that IL-17A is involved in several pathological tissue injuries. However, the function of IL-17A in BBB breakdown remains rarely discussed. Here, our study found that *E. coli*-induced IL-17A led to the degradation of tight junction proteins (TJs) and adherens junction proteins (AJs) in human brain microvascular endothelial cells (hBMECs) through inhibiting protease proteinase 3 (PRTN3)/protease-activated receptor 2 (PAR-2) axis, thus increasing the permeability of BBB. In summary, this study uncovered the involvement of IL-17A in regulating BBB integrity and proposed a novel regulatory mechanism, which could be potential therapeutic targets of *E. coli* meningitis.

## Introduction

Bacterial meningitis is a severe life-threatening infectious disease of the central nervous system (CNS) and a major cause of death or disability worldwide, especially in newborns (Leib and Tauber, 1999). There are a variety of pathogens that are classically associated with bacterial meningitis including Group B *Streptococcus*, *Streptococcus pneumoniae*, *Escherichia coli*, *Neisseria meningitidis*, and *Haemophilus influenzae* type B (Snyder and Brunjes, 1965; Booy and Kroll, 1998; Orihuela et al., 2009; Mook-Kanamori et al., 2011). Among these, meningitic *E. coli* is the most common Gram-negative bacillary organism (Janowski and Newland, 2017). To cause meningitis, *E. coli* must persist in the blood long enough, interact with and cross the blood–brain barrier (BBB), and invoke inflammatory responses (Kim, 2003).

Blood–brain barrier is a microvasculature that coordinates the movement of molecules and cells between the CNS and bloodstream (Zlokovic, 2008). It comprises brain microvascular endothelial cells (BMECs), pericytes, and astrocyte endfeet and maintains CNS homeostasis (Hawkins and Davis, 2005). Among these component cells, BMECs are the most direct and functional structural component of BBB and are characterized by the presence of tight junction proteins (TJs) and adherens junction proteins (AJs) (Dejana et al., 2009; Edwards et al., 2013). TJs are mainly composed of zonula occludens (ZOs), occludin, claudins, and AJs are primarily composed of vascular endothelial cadherin (VE-cadherin) (Bayir and Sendemir, 2021). The distribution or decrease of these TJs and AJs leads to an increased BBB permeability. Increased BBB permeability has been reported in numerous diseases, such as neoplasia, hypertension, experimental allergic encephalomyelitis, trauma, and neurotropic viral infections (Scholz et al., 2007; Candelario-Jalil et al., 2009). Inflammatory factors such as tumor necrosis factor alpha (TNF-α), interleukin-6 (IL-6), C-C motif ligand 2 (CCL2, also known as MCP-1) are reported to mediate BBB breakdown, which ultimately leads to the infiltration of peripheral leukocytes and brain injury (Sarami Foroshani et al., 2018; Siqueira et al., 2021; Xu D. et al., 2021). However, the underlying mechanisms by which these factors regulate BBB permeability in response to infection remain largely unclear.

Interleukin-17A (IL-17A) is the first identified member of the IL-17 family. Both αβT-cells and γδT-cells are associated with its production in infectious diseases (Papotto et al., 2017; Stockinger and Omenetti, 2017). Increased understanding of the biology of IL-17A has revealed that this inflammatory cytokine is involved in the modulation of acute or chronic bacterial infections, as well as other inflammation-associated diseases (Veldhoen, 2017). It has been shown that overproduced IL-17A promotes hyperinflammation and tissue damage in various diseases. For example, IL-17A mediates the production of vascular endothelial growth factor (VEGF), a major manager for vasculopathy, and aggravates neovascular retinopathy (Talia et al., 2016). Moreover, IL-17A also increases the permeability of alveolar epithelia (Bai et al., 2021). Notably, increasing studies support that IL-17A is involved in the breakdown of BBB integrity and subsequent neuroinflammation, for example, in multiple sclerosis (MS) or Group A *Streptococcus* infection (Tzartos et al., 2008; Dileepan et al., 2016). Despite these, whether IL-17A mediates BBB disruption in meningitic *E. coli* and the detailed mechanism by which IL-17A disrupts the BBB remain poorly understood.

In the present study, we characterized IL-17A as significantly up-regulated and an essential inflammatory cytokine in mouse brains in response to *E. coli*. Our *in vivo* and *in vitro* results demonstrated that IL-17A contributed to the disruption of BBB integrity by decreasing TJs and AJs. Further investigation suggested IL-17A down-regulated TJs and AJs of BMECs at the post-transcriptional level by inhibiting serine protease proteinase 3 (PRTN3)/protease-activated receptor 2 (PAR-2) axis. These observations indicated a novel strategy regarding the mechanism of meningitic *E. coli*-induced IL-17A in disrupting BBB and aggravating CNS dysfunction, which might be a potential therapeutic target for *E. coli* meningitis.

## Materials and Methods

### Bacterial Strains and Cell Culture

*Escherichia coli* strain PCN033 used herein was initially isolated from swine in cerebrospinal fluid from a diseased farm in China, 2006 (Liu et al., 2015). Bacterial cells were routinely grown in Luria-Bertani medium at 37 °C.

The human brain microvascular endothelial cells (hBMECs) were routinely cultured in RPMI1640 supplemented with 10% fetal bovine serum (FBS), 2 mM L-glutamine, 1 mM sodium pyruvate, essential amino acids, non-essential amino acids, vitamins, and penicillin and streptomycin (100 U/mL) in 37°C incubator under 5% CO_2_ until monolayer confluence (Stins et al., 2001). Confluent cells were washed with Hanks’ balanced salt solution and starved in serum-free medium (1:1 mixture of Ham’s F-12 and M-199) for 16–18 h before the experiment.

### Animal Infection Assay

The C57BL/6 wild-type mice and IL-17A knockout mice (kindly provided by Prof. Anding Zhang in Huazhong Agricultural University) were challenged with *E. coli* through the tail vein at 1 × 10^7^ CFUs suspended and diluted in phosphate-buffered saline (PBS; pH 7.4). The mice were anesthetized and the brains were harvested after cardiac perfusion for further assays.

For survival assay, anesthetized mice were pre-treated with IL-17A (10 ng/mouse, i.p.) or PBS 12 h before injection *E. coli*, followed by the injection (i.v.) of *E. coli*. The survival of each group of mice (*n* = 14) was recorded during the observation period of 24 h after E. coli infection.

### Evan’s Blue Assay

The BBB permeability of C57BL/6 wild-type mice and IL-17A knockout mice was evaluated using Evan’s blue dye (St. Louis, MO, United States), 500 μL Evan’s blue (5 mg/mL) was injected *via* the tail vein to allow circulation for 10 min before the mice were sacrificed and perfused. Brains were then taken and photographed for extravascular staining of the dye.

### Reverse Transcription and Real-Time Polymerase Chain Reaction

TRIzol reagent (Aidlab Biotech, Beijing, China) was utilized to isolate total RNA of brains or hBMECs. Aliquots (500 ng) of the total RNA in each sample were subjected to cDNA synthesis using the HiScript II Q RT SuperMix (Vazyme, Nanjing, China). Real-time PCR was performed with the real-time PCR thermal cycler qTOWER3 (Analytikjena, Jena, Germany) using MonAmp™ SYBR^®^ Green qPCR Mix (Monad Biotech, China) according to the manufacturers’ recommendations. Primers for real-time PCR were listed in Supplementary Table 1. Transcriptional levels of the target mRNA were normalized to GAPDH.

### Cytokine mRNA Assay

QuantiGene Plex 2.0 Assay (Panomics, Santa Clara, CA, United States) was used for cytokine mRNA quantification in the mouse brains as described elsewhere (Kottilil et al., 2009).

### Cytokine Protein Assay

Procartaplex Multiplex Immunoassays (eBioscience, San Diego, CA, United States) was used for measuring cytokines of brain lysates according to the manufacturer’s instructions (Lu et al., 2015).

### Western Blotting

Mouse brains or hBMECs were lysed using radioimmunoprecipitation assay buffer (EpiZyme, Shanghai, China) with protease inhibitor cocktail (MedChemExpress, Monmouth, NJ, United States), followed by centrifugation at 12,000 rpm for 15 min at 4°C to remove the insoluble cell debris. The protein concentrations were measured by using the bicinchoninic acid protein assay kit (CWBiotech, Beijing, China). Equal amounts of protein were further separated by 12% SDS-PAGE and electrophoretically transferred to polyvinylidene difluoride (PVDF) membranes. The blots were blocked with Tris-buffered saline-Tween (TBST) containing 5% bovine serum albumin for 2 h, and subsequently incubated with primary antibodies against ZO-1 (1:1,000; 220 kDa; Abcam, Cambridge, MA, United States), Occludin (1:1,000; 59 kDa; Abcam, Cambridge, MA, United States), Claudin-5 (1:1,000; 23 kDa; Affinity Biosciences, Changzhou, China), VE-Cadherin (1:1,000; 120 kDa; Affinity Biosciences, Changzhou, China), IL-17A (1:1,000; 18 kDa; Proteintech, Chicago, IL, United States), PRTN3 (1:1,000; 28 kDa; Proteintech, Chicago, IL, United States), β-actin (1:5,000; 42 kDa; Proteintech, Chicago, IL, United States). Membranes were subsequently washed and incubated with horseradish peroxidase-conjugated anti-rabbit or anti-mouse secondary antibodies (1:5,000; Biodragon, Beijing, China). The blots were visualized with the Super electrochemiluminescence Prime kit (US Everbright, Suzhou, China) and densitometrically analyzed using Image Lab software (Bio-Rad, Hercules, CA, United States).

### Histopathological Examination

For hematoxylin and eosin (H&E) assay, mice brain tissues were immersed in 4% paraformaldehyde for 4h, and transferred to 70% ethanol. Individual lobes of tissues biopsy material were placed in processing cassettes, dehydrated through a serial alcohol gradient, and embedded in paraffin wax blocks. Before staining, 5 μm-thick tissue sections were dewaxed in xylene, rehydrated through decreasing ethanol concentrations, and washed in PBS, and then stained with hematoxylin and eosin. After staining, sections were dehydrated through increasing concentrations of ethanol and xylene.

### Immunofluorescence Analysis

For immunofluorescence (IF) assay, sections were incubated with the primary antibody against ZO-1, Occludin (Abcam, Cambridge, MA, United States), Claudin-5, VE-Cadherin (Affinity Biosciences, Changzhou, China), followed by incubation with Cy3 conjugated secondary antibody. The same sections were then incubated with CD31 (Proteintech, Chicago, IL, United States) primary antibody, followed by incubation with FITC conjugated secondary antibody prior to the final nucleus staining with DAPI. Sections were photographed and analyzed using BX41 Microscopy (Olympus, Tokyo, Japan).

### Electrical Cell-Substrate Impedance Sensing

To evaluate the real-time alteration of the monolayer cell resistance, electrical cell-substrate impedance sensing (ECIS) system (Applied BioPhysics, Troy, MI, United States) was applied to compare the transendothelial electrical resistance (TEER) values in hBMECs with or without recombinant IL-17A protein seeded on the collagen-coated, gold-plate electrodes in 96-well chamber slides (96W1E+) as previously described. Two ECIS parameters, R (Ω), representing the electrical cell-cell contacts, and Rb (Ωcm2), representing paracellular barrier, were extracted from the continuously recorded impedance spectra to reflect the real-time changes of the monolayer barrier function.

### Statistics

All results are displayed as mean ± SD, and the significance of differences between groups was evaluated by one-way ANOVA. The survival curve analysis, log-rank (Mantel–Cox) was performed to test the significance of the difference between the evaluation groups. All statistical analyses were performed using GraphPad Prism software.

## Results

### Interleukin-17A Was Significantly Up-Regulated in *Escherichia coli*-Challenged Mice

**FIGURE 1:**
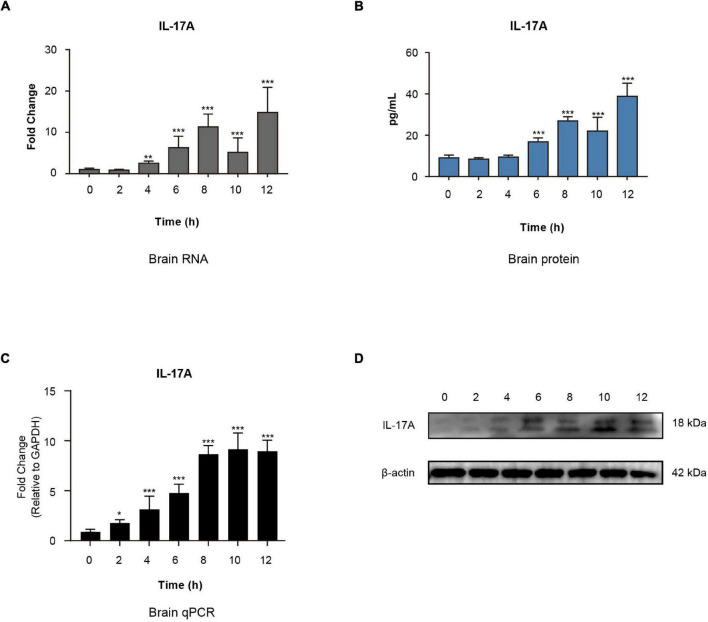
Interleukin-17A expression in mouse brains triggered by *E. coli* infection. Quantification of IL-17A expression in the brains of *E. coli* challenged mice at both the transcriptional and protein levels by applying QuantiGene Plex 2.0 Assay **(A)** or Procartaplex Multiplex Immunoassays **(B)**. **p < 0.01 and ***p < 0.001 by one-way ANOVA analysis. Data were collected and presented as mean ± SD from three replicates at each time point. **(C)** Real-time PCR verification of the IL-17A mRNA transcription in the brains of *E. coli* challenged mice. Data were collected and presented as mean ± SD from three replicates at each time point. *p < 0.05 and ***p < 0.001 by one-way ANOVA analysis. **(D)** Western blot verification of the IL-17A protein expression in the brains of *E. coli* challenged mice. β-Actin was used as the loading control.

To evaluate the intensity of inflammatory responses elicited by meningitic *E. coli*, the mRNA transcription, as well as protein expression of cytokines and chemokines, were assessed in the brain by using QuantiGene Plex 2.0 Multiplex assay and Procartaplex Multiplex Immunoassays. Among these data, IL-17A was significantly up-regulated within hours of infection at the transcriptional level (Figure 1A) and expression level (Figure 1B). By applying qPCR and Western blotting assay, we further verified that the expression of IL-17A in the brain was increased in a time-dependent manner (Figures 1C,D). These findings suggested that meningitic *E. coli* infection in mice can cause a significant increase of IL-17A in the brain.

### Interleukin-17A Promoted the Disruption of Blood–Brain Barrier Integrity

**FIGURE 2:**
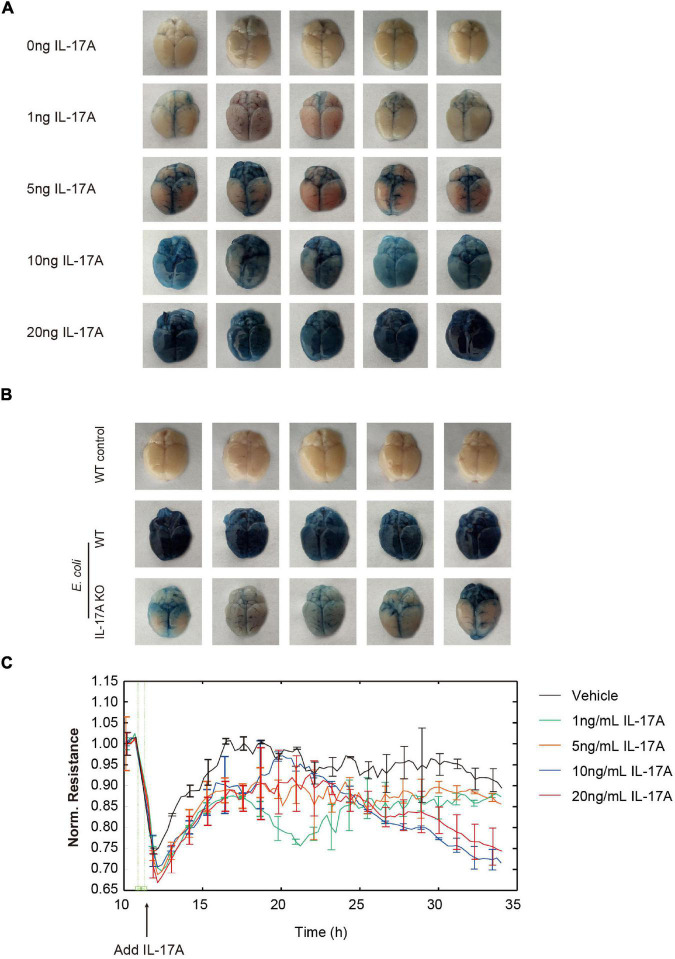
Interleukin-17A contributes to the increase of BBB permeability. **(A)** Effects of the recombinant mouse IL-17A (0, 1, 5, 10, and 20 ng/mouse) on the permeability of the mice brain evaluated by Evan’s blue approach. **(B)** The permeability of control mice, *E. coli* challenged WT or IL-17A KO mice evaluated by Evan’s blue approach. **(C)** TEER changes of hBMECs in the treatment of multiple dosage of IL-17A monitored by the ECIS system. Data were collected and presented as mean ± SD from three replicated wells at each time point.

Since a high level of IL-17A was detected in mouse brains after *E. coli* infection, we next investigated the function of IL-17A in regulating BBB permeability. As Figure 2A shown, intravenous (tail vein) injection of recombinant IL-17A (1, 5, 10, and 20 ng/mouse) for 12 h could dose-dependently increase the amount of Evans blue dye leaking out of the blood vessels compared with the control, which meant the increase of BBB permeability. In addition, IL-17A could also time-dependently increase the permeability of BBB (Supplementary Figure 2A). Further observation indicated that *E. coli*-caused increase of BBB permeability in WT mice could be restored by knocking out IL-17A (Figure 2B).

The hBMECs were subsequently used as *in vitro* model. We additionally tested the effects of IL-17A on TEER value, an important indicator of the monolayer permeability (Fu et al., 2021), by application of ECIS system. The results showed that IL-17A could dose-dependently down-regulate the TEER value of hBMECs monolayer (Figure 2C). Taken together, these observations indicated that *E. coli*-induced IL-17A could negatively affect the integrity of BBB.

### Interleukin-17A Disrupted Blood–Brain Barrier Integrity by Downregulating Tight Junction Proteins and Adherens Junction Proteins

**FIGURE 3:**
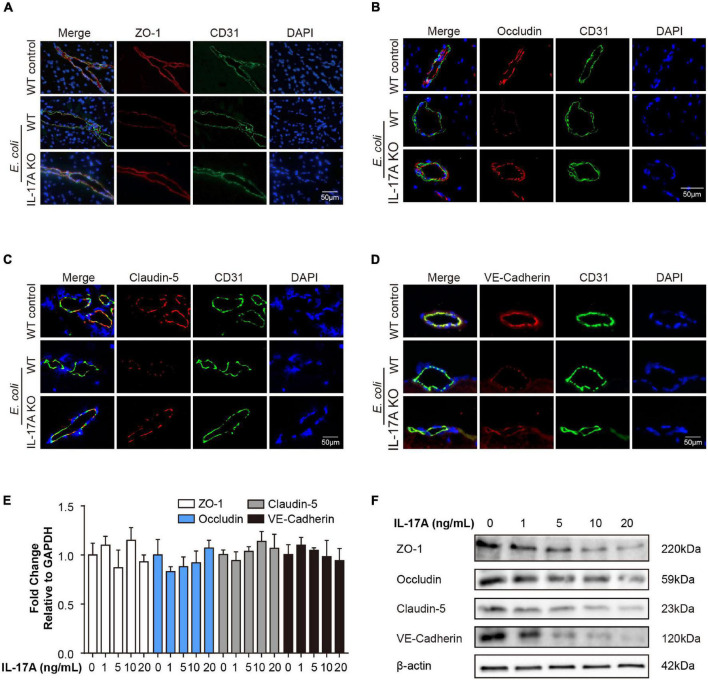
Interleukin-17A treatment downregulates the expression of TJs and AJs. IF analysis of vascular endothelium integrity in infected WT or IL-17A KO mice. ZO-1 **(A)**, Occludin **(B)**, Claudin-5 **(C)**, and VE-Cadherin **(D)** were selected as the markers reflecting the integrity of the vascular endothelium. CD31 was applied explicitly for labeling the microvessels. Scale bar indicates 50 μm. **(E)** The qPCR analysis of ZO-1, Occludin, Claudin-5, and VE-Cadherin transcription in hBMECs treated by multiple dosages of IL-17A (0, 1, 5, 10, and 20 ng/mL). GAPDH was used as the internal reference. Data were presented as mean ± SD from three independent experiments. **(F)** Western blot analysis of ZO-1, Occludin, Claudin-5, and VE-Cadherin in hBMECs in response to multiple dosages of IL-17A. β-Actin was used as the loading control.

As mentioned above, TJs and AJs determine the integrity of BBB, and we investigated whether TJs or AJs were involved in the IL-17A mediated disruption of BBB. *In vivo*, IF was performed to examine the distribution and expression of ZO-1, Occludin, Claudin-5, and VE-Cadherin in mouse brain tissues. The results showed that ZO-1, Occludin, Claudin-5, and VE-Cadherin were well-organized and distributed around the vascular in the control mice brain. In contrast, when treated with *E. coli*, the TJs and AJs around the vascular became inconsecutively distributed, irregular gapped, or down-regulated in WT mice. In contrast, these adverse effects of infection on TJs and AJs were significantly reversed by knocking-out IL-17A (Figures 3A–D).

*In vitro*, by treating hBMECs with different dosages of recombinant IL-17A, the expression of ZO-1, Occludin, Claudin-5, and VE-Cadherin at the protein level was significantly decreased. However, there was no significant difference at the transcriptional level (Figures 3E,F). These findings suggested that in meningitic *E. coli* infection, IL-17A was able to affect the integrity of BBB by down-regulating TJs and AJs at the post-transcriptional level.

### The Post-transcriptional Regulation of Interleukin-17A to Tight Junction Proteins and Adherens Junction Proteins Through Inhibiting Proteinase 3/Protease-Activated Receptor 2 Axis

**FIGURE 4:**
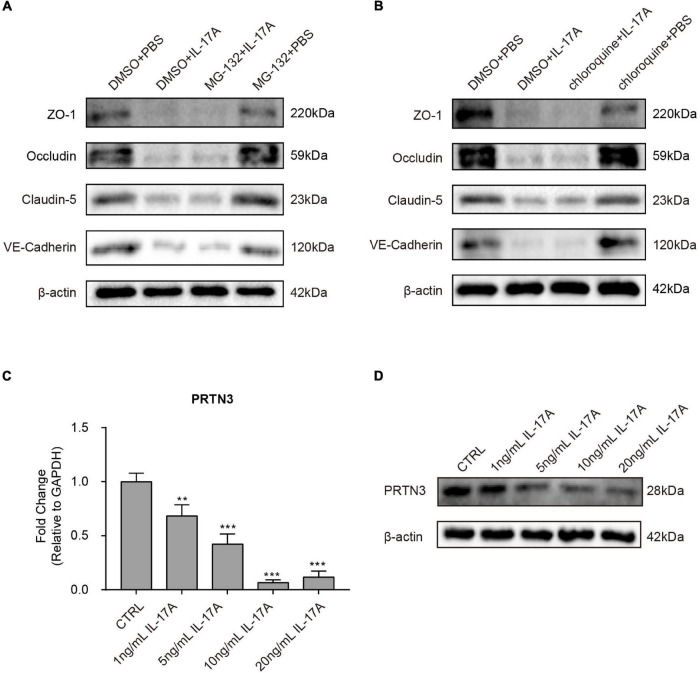
Interleukin-17A treatment decreases PRTN3 expression hBMECs. Western blot analysis of ZO-1, Occludin, Claudin-5, and VE-Cadherin extracted from IL-17A untreated hBMECs and IL-17A treated hBMECs with or without the presence of Proteasome inhibitor MG-132 **(A)** or autophagy inhibitor chloroquine **(B)**. β-Actin was used as the loading control. **(C)** The qPCR analysis of PRTN3 transcription in hBMECs treated by multiple dosages of IL-17A (0, 1, 5, 10, and 20 ng/mL). GAPDH was used as the internal reference. Data were presented as mean ± SD from three independent experiments. **p < 0.01 and ***p < 0.001 by one-way ANOVA analysis. **(D)** Western blot analysis of PRTN3 in hBMECs in response to multiple dosages of IL-17A. β-Actin was used as the loading control.

As we have demonstrated that IL-17A could post-transcriptionally regulate the expression of TJs and AJs, further investigations were taken to determine a more detailed regulatory mechanism of this phenomenon. It was reported that the ubiquitin-proteasome system and autophagy were the primary mechanisms for degrading protein; however, neither the proteasome inhibitor MG-132 nor the autophagy inhibitor chloroquine showed reverse decrease of TJs and AJs in hBMECs induced by IL-17A (Figures 4A,B).

Previous studies have demonstrated that a serine protease PRTN3 was able to enhance endothelial cell barrier and thus vascular integrity through cleaving and activating PAR-2 (Kuckleburg and Newman, 2013). Therefore, we next focused on the expression of PRTN3, and the observation showed that the mRNA transcription (Figure 4C) and protein expression (Figure 4D) of PRTN3 were significantly down-regulated in hBMECs in response to IL-17A treatment in a dose-dependent manner.

**FIGURE 5:**
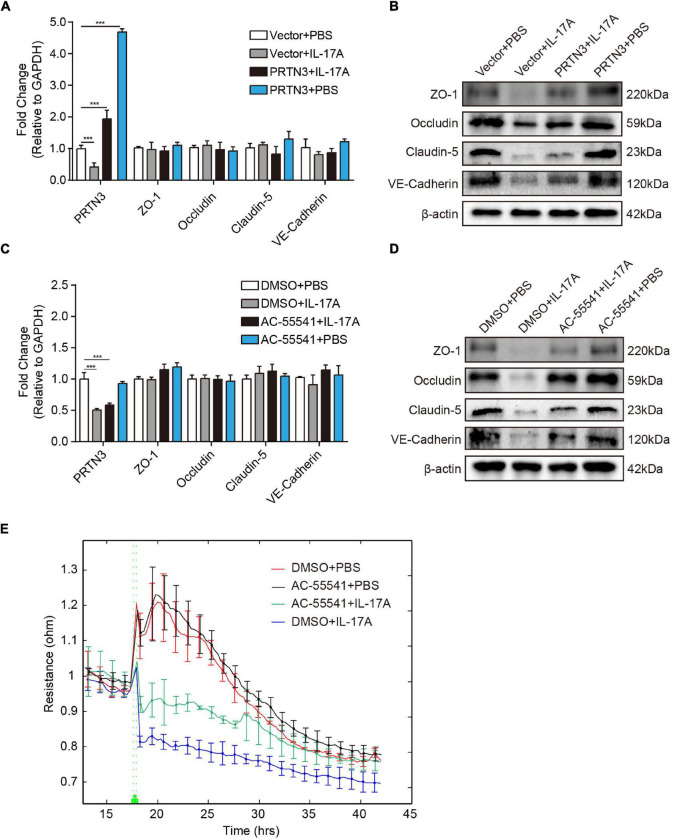
Interleukin-17A disrupts TJs and AJs by inhibiting PRTN3/PAR-2 axis. **(A)** The qPCR analysis of ZO-1, Occludin, Claudin-5, and VE-Cadherin extracted from IL-17A untreated hBMECs and IL-17A treated hBMECs with or without the overexpression of PRTN3. GAPDH was used as the internal reference. Data were presented as mean ± SD from three independent experiments. ***p < 0.001 by one-way ANOVA analysis. **(B)** Western blot of ZO-1, Occludin, Claudin-5 and VE-Cadherin extracted from IL-17A untreated hBMECs and IL-17A treated hBMECs with or without overexpression of PRTN3. β-Actin was used as the loading control. **(C)** The qPCR analysis of ZO-1, Occludin, Claudin-5, and VE-Cadherin extracted from IL-17A untreated hBMECs and IL-17A treated hBMECs with or without PAR-2 agonist AC-55541. GAPDH was used as the internal reference. Data were presented as mean ± SD from three independent experiments. ***p < 0.001 by one-way ANOVA analysis. **(D)** Western blot of ZO-1, Occludin, Claudin-5, and VE-Cadherin extracted from IL-17A untreated hBMECs and IL-17A treated hBMECs with or without PAR-2 agonist AC-55541. β-Actin was used as the loading control. **(E)** TEER changes of the IL-17A untreated hBMECs and IL-17A treated hBMECs with or without PAR-2 agonist AC-55541 monitored by the ECIS system. Data were collected and presented as mean ± SD from three replicated wells at each time point.

To examine whether the decrease of PRTN3 reduces the barrier function of hBMECs, PRTN3 overexpression constructs were used to validate its regulative effects on TJs. As demonstrated, PRTN3 overexpression did not influence the mRNA transcription of the ZO-1, Occludin, Claudin-5, and VE-Cadherin (Figure 5A). Meanwhile, at the protein level, PRTN3 overexpression could significantly restore the decrease of ZO-1, Occludin, Claudin-5, and VE-Cadherin in hBMECs caused by IL-17A (Figure 5B). As mentioned above, PRTN3 enhances the endothelial cell barrier through activating PAR-2; we subsequently determined the function of PAR-2 in TJs and AJs disruption. We pre-treated hBMECs with AC-55541, a novel small molecule agonist of PAR-2. The results revealed that PAR-2 agonist significantly inhibited the decrease of TJs and AJs protein expression induced by IL-17A (Figure 5D), and it also had no effect on the transcription of TJs and AJs (Figure 5C). Correspondingly, the ECIS assay showed that PAR-2 agonist could ameliorate the loss of TEER value of hBMECs monolayer induced by IL-17A (Figure 5E). These results indicated that IL-17A could decrease the expression of TJs and AJs of hBMECs at the post-transcriptional level *via* inhibiting PRTN3/PAR-2 axis, thus augmented the vascular permeability of BBB.

### Interleukin-17A Aggravated Brain Tissue Damage and Reduced Survival Rate in *Escherichia coli*-Challenged Mice

**FIGURE 6:**
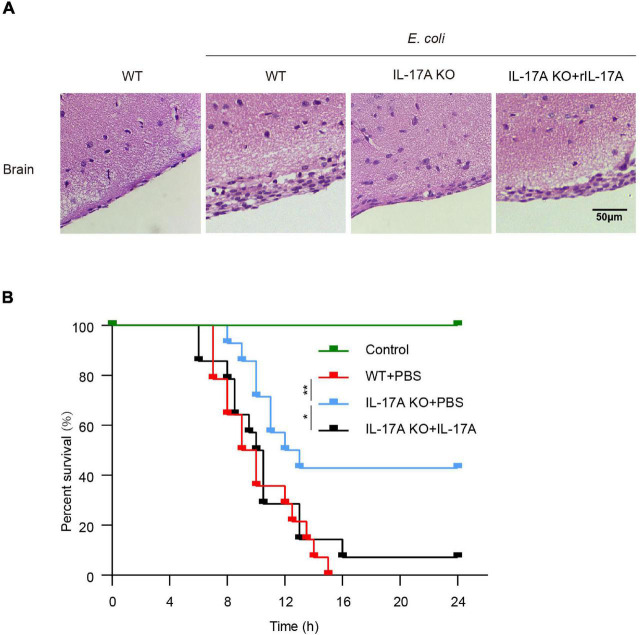
Interleukin-17A aggravated brain tissue damage and reduced survival rate in *E. coli*-infected mice. **(A)** WT mice, IL-17A KO mice with or without adding recombinant IL-17A, were infected with *E. coli*, the histopathological changes in the brain were investigated by H&E staining. Scale bar indicates 50 μm. **(B)** Survival analysis curves for WT mice, IL-17A KO mice with or without adding recombinant IL-17A infected with *E. coli*. Data were collected and shown as Kaplan–Meier survival curves from 14 individual mice. *p < 0.05 and **p < 0.01.

We further determined the effects of IL-17A in mice throughout *E. coli* meningitis pathogenesis. Histologic sections of the brain showed that *E. coli* infection in WT mice could induce tissue damage such as meningeal thickening and hemorrhage. This pathological phenomenon was severer than that in IL-17A KO mice. Notably, we observed that this phenomenon could be well restored by the addition of recombinant IL-17A (Figure 6A).

Based on these observations, we evaluated the effects of IL-17A on the lethality of mice. As shown in Figure 6B, when challenged with *E. coli*, mice with IL-17A knocked out had a higher survival rate than WT mice. In contrast, IL-17A KO mice pre-injected with recombinant IL-17A could not improve the survival rate of the infection. These data suggested IL-17A aggravated the damage to the brain and thus led to increased mortality.

## Discussion

The BBB is a highly specialized structure that maintains CNS homeostasis (Tajes et al., 2014). In bacterial meningitis, pathogens such as *E. coli*, *Neisseria*, and Group B *Streptococcus* always penetrate BBB, induce local CNS inflammatory responses, and lead to the disruption of BBB (Booy and Kroll, 1998; Kim, 2000; Herbert et al., 2004). BBB disruption is a typical pathological phenomenon in many diseases related to CNS disorders, including bacterial meningitis, Japanese encephalitis, ischemic stroke, etc. (Wang et al., 2018; Liu C. et al., 2020; Xu B. et al., 2021). Accumulating evidence has suggested that host factors and cytokines are involved in the regulation of TJs expression and contribute to BBB dysfunction. For example, cytokines such as TNF-α and IL-6 (Sarami Foroshani et al., 2018; Siqueira et al., 2021), chemokines such as CCL2 and IP-10 (Wang et al., 2018; Xu D. et al., 2021), and growth factors such as VEGF-A and PDGF-BB (Yang et al., 2016, 2019) are all reported to induce BBB breakdown. However, there are limited reports on whether IL-17A directly impacts BBB permeability. In the current study, we reported that, as an essential pro-inflammatory cytokine, IL-17A was significantly increased in mouse brains after meningitic *E. coli* PCN033 infection. With further *in vivo* and *in vitro* verification in response to infection, we revealed that meningitic *E. coli*-induced IL-17A significantly down-regulated the expression of TJs and AJs of hBMECs at the post-transcriptional level through inhibiting PRTN3/PAR-2 axis, thus augmenting endothelial permeability and disrupting BBB integrity. It ultimately facilitates brain damage and promotes the death of mice.

Interleukin-17A is a key inflammatory factor that contributes to the occurrence and development of several pathogenic injuries such as severe intestinal injury, pancreatic injury, and acute kidney injury (Chan et al., 2014; Kim et al., 2015). In *Bacteroides* infection, IL-17A is significantly increased and helps disrupt the intestinal barrier (Liu W. et al., 2020). In acute necrotizing pancreatitis, the large number of induced IL-17A is correlated significantly with TJs reduction and pancreatic injury (Guo et al., 2019). It has also been reported that IL-17A harms proximal tubule epithelium integrity and mediates renal injury (Dudas et al., 2011). As for the brain, our *in vivo* and *in vitro* data confirmed that IL-17A increases the permeability of BBB by down-regulating the expression of TJs at the protein level rather than the transcription level. To clarify its mechanism, many genes reported to be involved in BBB breakdown were validated by us. It was widely agreed that matrix metalloproteinases-2 (MMP-2), MMP-3, and MMP-9 are involved in the degradation of TJs (Rosenberg and Yang, 2007; Oppenheim et al., 2013; Weekman and Wilcock, 2016), however, in our study, IL-17A did not up-regulate the expression of these MMPs (Supplementary Figure 1). Furthermore, the ubiquitin-proteasome system and autophagy are considered the important pathways for protein degradation (Varshavsky, 2017). However, the results showed that these pathways were not involved in the IL-17A-induced degradation of TJs. It reminds us that IL-17A affects BBB permeability through a novel pathway.

Interestingly, it was reported that a serine protease called PRTN3 could enhance endothelial barrier function and thus vascular integrity by cleaving and activating PAR-2, a cathepsin S cleaves protease-activated receptor (Kuckleburg and Newman, 2013). Our *in vitro* studies also found that IL-17A caused a reduction of PRTN3 in both time-dependent and dose-dependent manners (Supplementary Figures 2B,C), while the application of PRTN3 overexpression construct or PAR-2 agonist can well restore the reduction of TJs and AJs caused by IL-17A, suggesting that IL-17A negatively regulates the BBB function at the post-transcriptional level through inhibiting PRTN3/PAR2.

Dysfunction of BBB has been reported to cause severe neurological complications, such as electrolyte disturbance, intracerebral hemorrhage, and increased intracranial pressure, all of which ultimately result in death (Duan et al., 2020; Yang et al., 2021). When challenged with meningitic *E. coli*, due to the BBB disruption induced by IL-17A, WT mice or IL-17A pre-treated KO mice suffered more severe brain injuries, such as hemorrhage and meningeal thickening compared to IL-17A KO mice. This pathological change accelerated the death process of mice and increased the mortality of mice. Nonetheless, it is increasingly recognized that inflammation is a double-edged sword during infection, which can be both a destroyer of tissue damage and a helper of eliminating infection (Chaudhry et al., 2013). For example, TNF-α was reported to trigger a sustained inflammation response to destroy the invading of *Mycobacterium tuberculosis* (MTB). However, excessive TNF-α can cause tissue damage and necrosis, thus giving rise to organ dysfunction (Mootoo et al., 2009). Our other study found that IL-17A might mediate *E. coli* clearance *via* increasing antimicrobial peptides, which require further investigation to comprehensively interpret the function of IL-17A throughout *E. coli* meningitis pathogenesis.

## Conclusion

In conclusion, the evidence highlights the importance of IL-17A in meningitic *E. coli*-induced BBB disruption. We demonstrate that IL-17A mediates TJs and AJs breakdown, thereby augmenting BBB permeability *via* inhibiting PRTN3/PAR2 axis during meningitic *E. coli* infection, leading to severe neuroinflammation and neuronal injury. Elucidating mechanisms of the IL-17A-induced BBB disruption may provide an accurate and effective target for preventing BBB breakdown in meningitis.

## Data Availability Statement

The original contributions presented in the study are included in the article/Supplementary Material, further inquiries can be directed to the corresponding author.

## Ethics Statement

The animal study was reviewed and approved by the Institutional Animal Care and Use Committee of Huazhong Agricultural University.

## Author Contributions

XW, BX, and JC designed the study, performed the experiments, and drafted the manuscript. JF, RY, and BY helped acquire data and process samples. BX and DH analyzed and interpreted the data. CT and HC provided technical support. XW and RY revised the manuscript. All authors have read and approved the final version of this article.

## Conflict of Interest

The authors declare that the research was conducted in the absence of any commercial or financial relationships that could be construed as a potential conflict of interest.

## Publisher’s Note

All claims expressed in this article are solely those of the authors and do not necessarily represent those of their affiliated organizations, or those of the publisher, the editors and the reviewers. Any product that may be evaluated in this article, or claim that may be made by its manufacturer, is not guaranteed or endorsed by the publisher.

## Supplementary Material

Supplementary Figure 1 The qPCR analysis of MMP2 **(A)**, MMP3 **(B)**, MMP9 **(C)**, and MMP12 **(D)** transcription in hBMECs treated by multiple dosages of IL17A (0, 1, 5, 10, and 20 ng/mL). GAPDH was used as the internal reference. Data were presented as mean ± SD from three independent experiments.

Supplementary Figure 2 The time course of the effects of IL-17A on BBB permeability *in vivo* and *in vitro*. **(A)** IL-17A time-dependently increased the permeability of BBB evaluated by Evan’s blue approach. **(B)** The transcription of PRTN3 in hBMECs treated with IL-17A for 3, 6, 9, and 12 h. GAPDH was used as the internal reference. Data were presented as mean ± SD from three independent experiments. ***p* < 0.01 and ****p* < 0.001 by one-way ANOVA analysis. **(C)** Western blot analysis of PRTN3 in hBMECs treated with IL-17A for 3, 6, 9, and 12 h. β-Actin was used as the loading control.

Supplementary Table 1 Overview of used RT-qPCR primer sequences.
