# Supplementary material for: Meningitic Escherichia coli-Induced Interleukin-17A Facilitates Blood–Brain Barrier Disruption via Inhibiting Proteinase 3/Protease-Activated Receptor 2 Axis
Source: Front Cell Neurosci. 2022 Feb 11;16:814867. doi: 10.3389/fncel.2022.814867 (PMC8873187; doi:10.3389/fncel.2022.814867)

Supplementary Material

# Supplementary Table S1 Overview of used RT-qPCR primer sequences.

| Gene | Species | Forward sequence (5’-3’) | Reverse sequence (5’-3’) |
| --- | --- | --- | --- |
| *Gapdh* | Mouse | TGGATTTGGACGCATTGGTC | TTTGCACTGGTACGTGTTGAT |
| *Il17a* | Mouse | TTTAACTCCCTTGGCGCAAAA | CTTTCCCTCCGCATTGACAC |
| *GAPDH* | Human | CAACAGCCTCAAGATCATCAG | GAGTCCTTCCACGATACCA |
| *PRTN3* | Human | CACTGCCTGCGGGACATAC | TTCAGAAACACCTGAGCCACC |
| *ZO1* | Human | GACTTAAAGCTGCCTCAACAGA | GGTTTGTTTCAGGCGAAAGG |
| *OCLN* | Human | TTAACTTCGCCTGTGGAT | AGTGATCTTGCTCTGTTCT |

**Supplementary Figure S1** IL-17A had no effect on the expression of MMPs in hBMECs.


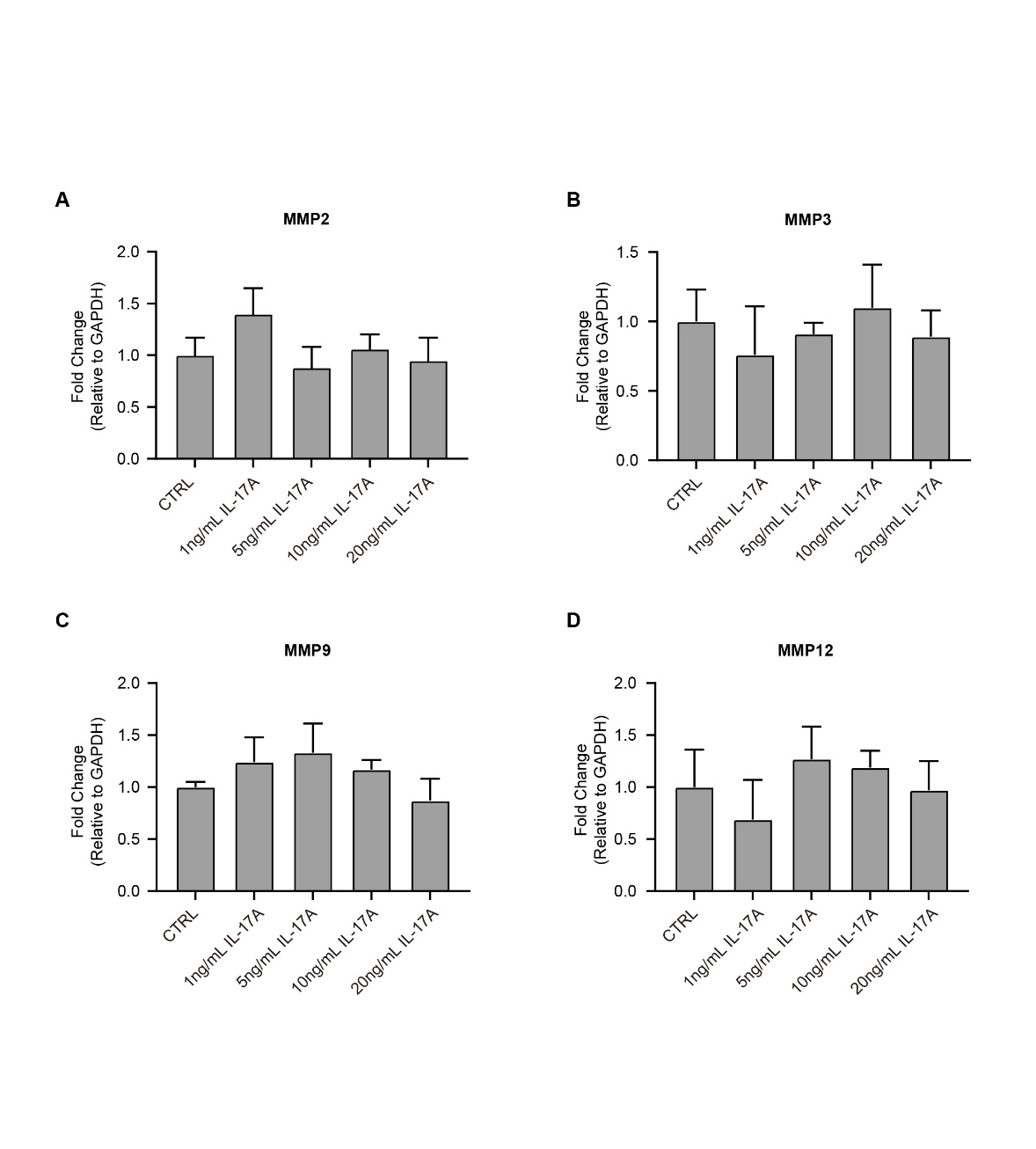


**Supplementary Figure S2** The time course of the effects of IL-17A on BBB permeability in vivo and in vitro.


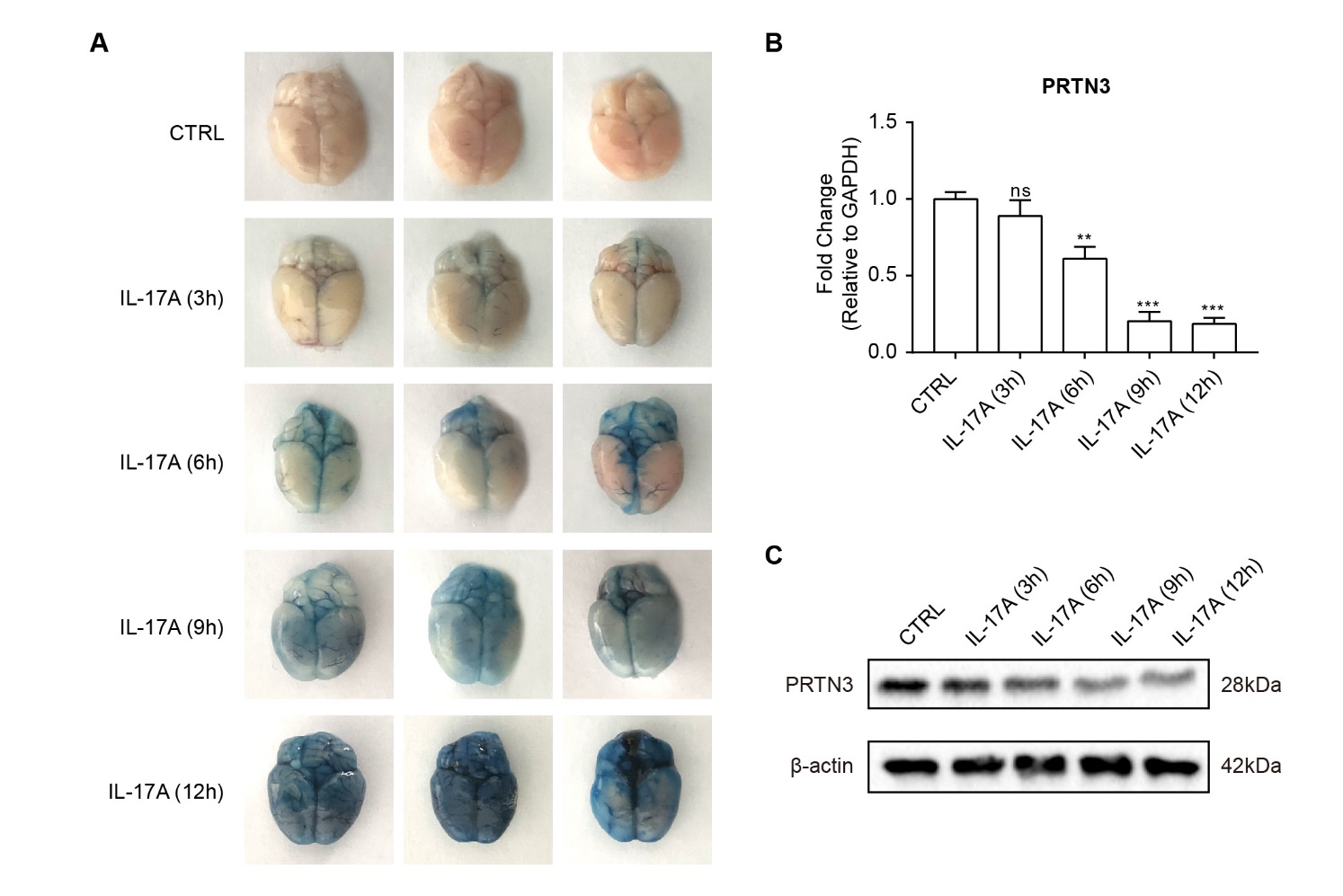

Supplement: Supplementary Figure 1 — The qPCR analysis of MMP2 (A), MMP3 (B), MMP9 (C), and MMP12 (D) transcription in hBMECs treated by multiple dosages of IL17A (0, 1, 5, 10, and 20 ng/mL). GAPDH was used as the internal reference. Data were presented as mean ± SD from three independent experiments. [file Data_Sheet_1.docx]
